# Artificial intelligence in rheumatology: a cross-sectional Scopus-based analysis

**DOI:** 10.1007/s00296-026-06182-5

**Published:** 2026-06-30

**Authors:** Dinmukhammed Otebay, Gulnara Kapanova, Bekaidar Nurmashev, Maidan Mukhamediyarov, Burhan Fatih Kocyigit

**Affiliations:** 1 Department of Public Health, Asfendiyarov Kazakh National Medical University, Almaty, Kazakhstan; 2 Department of Health Policy and Organization, Al-Farabi Kazakh National University, Almaty, Kazakhstan; 3 Department of Chemical Disciplines, Biology and Biochemistry, South Kazakhstan Medical Academy, Shymkent, Kazakhstan; 4 Department of Physical Medicine and Rehabilitation, Adana City Research and Training Hospital, University of Health Sciences, Adana, Türkiye

**Keywords:** Artificial intelligence, Rheumatology, Rheumatic diseases, Bibliometrics, Bibliometric analysis, Surveys and questionnaires

## Abstract

Artificial intelligence (AI) is increasingly used in clinical medicine. Rheumatology is well-suited to AI applications due to diagnostic complexity of rheumatic diseases, variable disease presentations, and multisystem involvement. This cross-sectional study examines global AI research in rheumatology through bibliometric analysis of Scopus data. The Scopus database was searched on May 5, 2026, using the terms "artificial intelligence" AND "rheum*" in the title, abstract, and keyword fields. No temporal restrictions were applied, and all English documents were analyzed. Bibliometric data, including publication year, country, institution, author, journal, and keywords, were extracted. Retracted articles were identified using Scopus tags and were manually verified through related journal editorial notices. Disease-specific publication counts were determined through independent searches for rheumatic diseases. Survey-based studies were identified through manual review. Linear regression analysis was conducted to assess temporal trends. A total of 1,057 publications were identified. Annual output remained below 10 until 2018, then rose sharply to a peak of 282 in 2025. Linear regression confirmed a significant upward trend (p < 0.001). The United States produced the most publications (n = 249), followed by the United Kingdom (n = 141) and India (n = 138). Harvard Medical School was the leading institution (n = 33), and Knitza, J., was the most prolific author (n = 17). *Rheumatology International* published the most articles (n = 27). Osteoarthritis (n = 1,017) and rheumatoid arthritis (n = 646) dominated the field, while systemic vasculitis (n = 40), Behçet disease (n = 29), and familial Mediterranean fever (n = 16) were underrepresented. Eleven survey-based studies were identified, mostly published between 2024 and 2025. Two articles were retracted due to peer review irregularities. AI research in rheumatology has increased significantly, with research outputs stemming from a limited number of countries, institutions, and disease categories. Osteoarthritis and rheumatoid arthritis are the most frequently explored diseases in the field. The increasing number of survey-based studies indicates heightened attention to clinician and patient perspectives. Future research should focus on expanding international collaboration, addressing gaps in AI research on underrepresented diseases, and strengthening ethical and methodological standards to facilitate broader AI adoption in rheumatology.

## Introduction

Artificial intelligence (AI) refers to the theoretical foundation and development of computer systems capable of performing tasks traditionally associated with human intelligence, such as speech recognition, learning, visual perception, mathematical computation, reasoning, problem-solving, decision-making, and language translation [1]. This concept is well established in the medical literature, and its integration into clinical practice has increased significantly in recent years [2]. Generative AI is a methodology that identifies patterns and distributions by analyzing training samples, thereby enabling the large-scale generation of realistic content across media such as text, images, audio, and video [3].

The introduction of ChatGPT by OpenAI in late 2022 marked a pivotal shift in the application of AI within medicine. Leveraging large language models, ChatGPT has positioned itself as one of the fastest-growing technological applications [4]. In the healthcare sector, this tool significantly broadened access to AI for clinicians and patients alike, enabling diverse applications such as clinical decision support, medical documentation, and patient education [5–7].

Rheumatology is well-suited to AI applications due to the diagnostic challenges posed by complex disease processes, variable clinical presentations, and multisystem involvement. Persistent diagnostic delays, difficulties with predicting treatment outcomes, and limited objective measures of disease activity highlight the potential value of AI in this field [8, 9]. In this context, AI may contribute to rheumatology by enabling early and accurate diagnosis, predicting disease activity and prognosis, modeling epidemiological trends, personalizing patient education, optimizing rehabilitation planning, and supporting telehealth and remote diagnostics [10–12].

This cross-sectional study analyzes the current state of AI research in rheumatology, focusing on geographical distribution, institutional and author productivity, disease-specific publication profiles, and temporal development trends, using Scopus-based bibliometric methods. The analysis identifies the most productive countries, institutions, and authors, and examines publication trends, journal distribution, and keyword patterns. Retracted publications and survey-based studies are also evaluated, and the role of AI in the literature is assessed for specific rheumatic diseases.

## Methods

The Scopus database was used to analyze scholarly publications concerning AI in rheumatology. Publication data were retrieved from searches conducted on May 5, 2026. The search strategy incorporated the terms "artificial intelligence" AND "rheum*" within the title, abstract, and keyword fields (TITLE–ABSTRACT–KEYWORDS). The asterisk (*) wildcard following "rheum" captured all relevant derivatives, including rheumatology, rheumatoid, rheumatic, and rheumatism, thereby broadening the search scope. No temporal restrictions were applied; all publications from the earliest available date through May 2026 were included. All document types indexed in Scopus, such as articles, reviews, and conference papers, were considered. Only English publications were included [13, 14].

The search strategy was implemented to ensure comprehensive coverage of the literature on the subject, minimize the risk of omitting relevant studies, and systematically assess the historical development of AI research in rheumatology. This approach sought to deliver a thorough overview of the topic and to rigorously evaluate the identified publications.

### Data collection

 For each retrieved publication, the title, authors, publication year, journal, institution, country, and keywords were recorded. Retracted papers were identified and documented.The reason for each retraction was manually documented by reviewing the corresponding journal editorial notices [15].

The ten most productive countries, institutions, authors, and journals were identified and ranked. The ten most frequently used keywords were reported, and keyword analysis was performed to assess thematic emphases within the field. Separate searches were conducted for rheumatoid arthritis, osteoarthritis, systemic lupus erythematosus, systemic sclerosis, psoriatic arthritis, spondyloarthritis, axial spondyloarthritis, Sjögren's syndrome, gout, myositis, fibromyalgia, systemic vasculitis, Behçet disease, and familial Mediterranean fever. Publication counts were compared and ranked for each category. An independent Scopus search was conducted using the search criteria "artificial intelligence" and "[rheumatic disease]." The number of publications retrieved was recorded. Survey-based studies were identified through manual review. The titles, abstracts, and keywords of all publications were examined, and the full text was reviewed when necessary to confirm the use of survey methodology. For each selected survey study, the title, publication year, target population, journal of publication, and citation count were recorded.

### Statistical analyses

Data were reported as counts (n). Temporal trends in annual publication output were assessed using linear regression, analyzed with the Statistical Package for the Social Sciences (SPSS), version 20.0 (SPSS Inc., Chicago, IL, USA). Statistical significance was set at p < 0.05.

## Results

### Annual publication trends

**Fig. 1:**
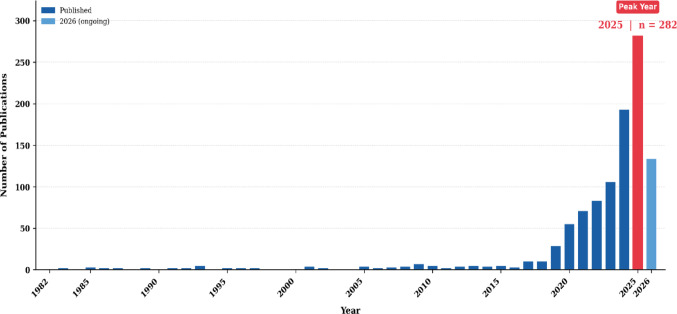
Annual publication trends in artificial intelligence and rheumatology research

The first paper on AI and rheumatology was published in 1982. Over the next four decades, fewer than 10 publications were published each year until 2018. Research activity in the field increased significantly from 2019, peaking at 282 publications in 2025 (Fig. 1). Linear regression analysis demonstrated a statistically significant increase in annual publication counts during the research period (slope = 2.49 publications/year, R^2^ = 0.343, p < 0.001). These results indicate a notable rise in research at the intersection of AI and rheumatology, reflecting the growing use of AI in rheumatological studies. Data from 2026, which represented only half a year, were excluded from the regression model. In total, 1,057 articles were identified during the research period.

### Country-based data

**Fig. 2:**
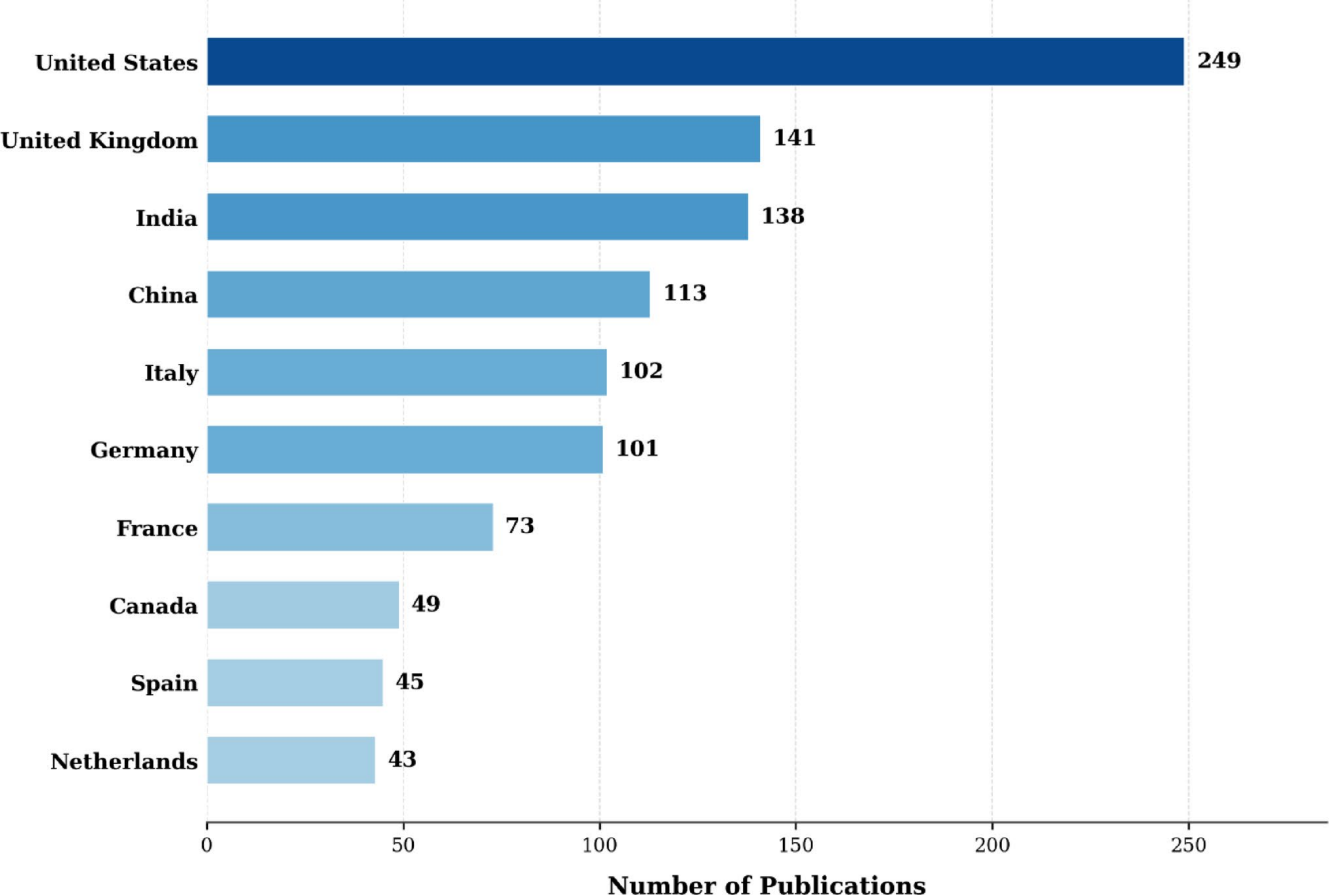
Top 10 most productive countries in artificial intelligence and rheumatology research

Ten countries produced most of the articles on AI and rheumatology. The United States led with 249 publications, followed by the United Kingdom (n = 141) and India (n = 138). China ranked fourth with 113 publications, while Italy (n = 102) and Germany (n = 101) had similar outputs. (Fig. 2).

### Geographic distribution—selected countries

Upon analyzing specific nations as representatives of diverse research engagement, Türkiye (n = 41), Japan (n = 28), and South Korea (n = 20) were comparatively prolific contributors to the field. Poland produced 15 articles, reflecting a moderate level of participation. Ukraine published 5 articles, while Kazakhstan only 3. Uzbekistan, Kyrgyzstan, and Tajikistan had no publications in the dataset.

### Most productive institutions

Harvard Medical School led with 33 publications, followed by Inserm with 28 and Brigham and Women's Hospital with 25. German institutions also ranked highly, with Friedrich-Alexander-Universität Erlangen-Nürnberg (n = 24), Charité – Universitätsmedizin Berlin (n = 22), and Universitätsklinikum Erlangen (n = 20) all in the top six. The University of Manchester, University College London, and Imperial College London produced 17, 17, and 16 publications, respectively. The University of Bari Aldo Moro contributed 17 publications to Italy. Table 1 lists the 10 most productive institutions.

**Table 1:** Top 10 most productive institutions in artificial intelligence and rheumatology research

Rank	Institution	Number of Publications
1	Harvard Medical School	33
2	Inserm	28
3	Brigham and Women's Hospital	25
4	Friedrich-Alexander-Universität Erlangen-Nürnberg	24
5	Charité-Universitätsmedizin Berlin	22
6	Universitätsklinikum Erlangen	20
7	The University of Manchester	17
8	University College London	17
9	Università degli studi di Bari Aldo Moro	17
10	Imperial College London	16

### Most prolific authors

Knitza, J., was the most prolific author with 17 publications, followed by Venerito, V., with 14, and Gupta, L., with 12. Krusche, M., Kleyer, A., and Hügle, T. each published 11 papers. Simon, D., and Schett, G. produced 10 publications each, while Ravindran, V., and Iannone, F. each had 8. Table 2 lists the ten most productive authors and their publication counts.

**Table 2:** Top 10 most prolific authors in artificial intelligence and rheumatology research

Rank	Author	Number of Publications
1	Knitza, J	17
2	Venerito, V	14
3	Gupta, L	12
4	Krusche, M	11
5	Kleyer, A	11
6	Hügle, T	11
7	Simon, D	10
8	Schett, G	10
9	Ravindran, V	8
10	Iannone, F	8

### Keyword analysis

Analysis of keyword frequency showed that Artificial Intelligence was the most common term (n = 929), followed by Human (n = 842) and Rheumatoid Arthritis (n = 553). Humans appeared 474 times, highlighting variability in singular and plural indexing. Article (394) and Machine Learning (320) ranked fifth and sixth, reflecting the literature’s methodological focus. Rheumatology (255) and Review (250) followed, while Female (236) and Male (225) completed the top ten (Table 3).

**Table 3:** Top 10 most frequently used keywords in artificial intelligence and rheumatology research

Rank	Keyword	Number
1	Artificial Intelligence	929
2	Human	842
3	Rheumatoid Arthritis	553
4	Humans	474
5	Article	394
6	Machine Learning	320
7	Rheumatology	255
8	Review	250
9	Female	236
10	Male	225

### Journal-based data

*Rheumatology International* published the most articles in the field (27), followed by *Frontiers in Medicine* and *Frontiers in Immunology* (each with 22). *Clinical Rheumatology* had 18 publications. *The Journal of Rheumatology* and *Annals of the Rheumatic Diseases* each published 16 papers. *Rheumatology (United Kingdom*) and *Diagnostics* each contributed 15; *RMD Open* published 14; and *Therapeutic Advances in Musculoskeletal Disease* had 13. Table 4 lists the 10 most prolific journals.

**Table 4:** Top 10 most productive journals in artificial intelligence and rheumatology research

Rank	Journal	Number of Publications
1	*Rheumatology International*	27
2	*Frontiers in Medicine*	22
3	*Frontiers in Immunology*	22
4	*Clinical Rheumatology*	18
5	*The Journal of Rheumatology*	16
6	*Annals of the Rheumatic Diseases*	16
7	*Rheumatology (United Kingdom)*	15
8	*Diagnostics*	15
9	*RMD Open*	14
10	*Therapeutic Advances in Musculoskeletal Disease*	13

### Disease-specific distribution of publications

**Fig. 3:**
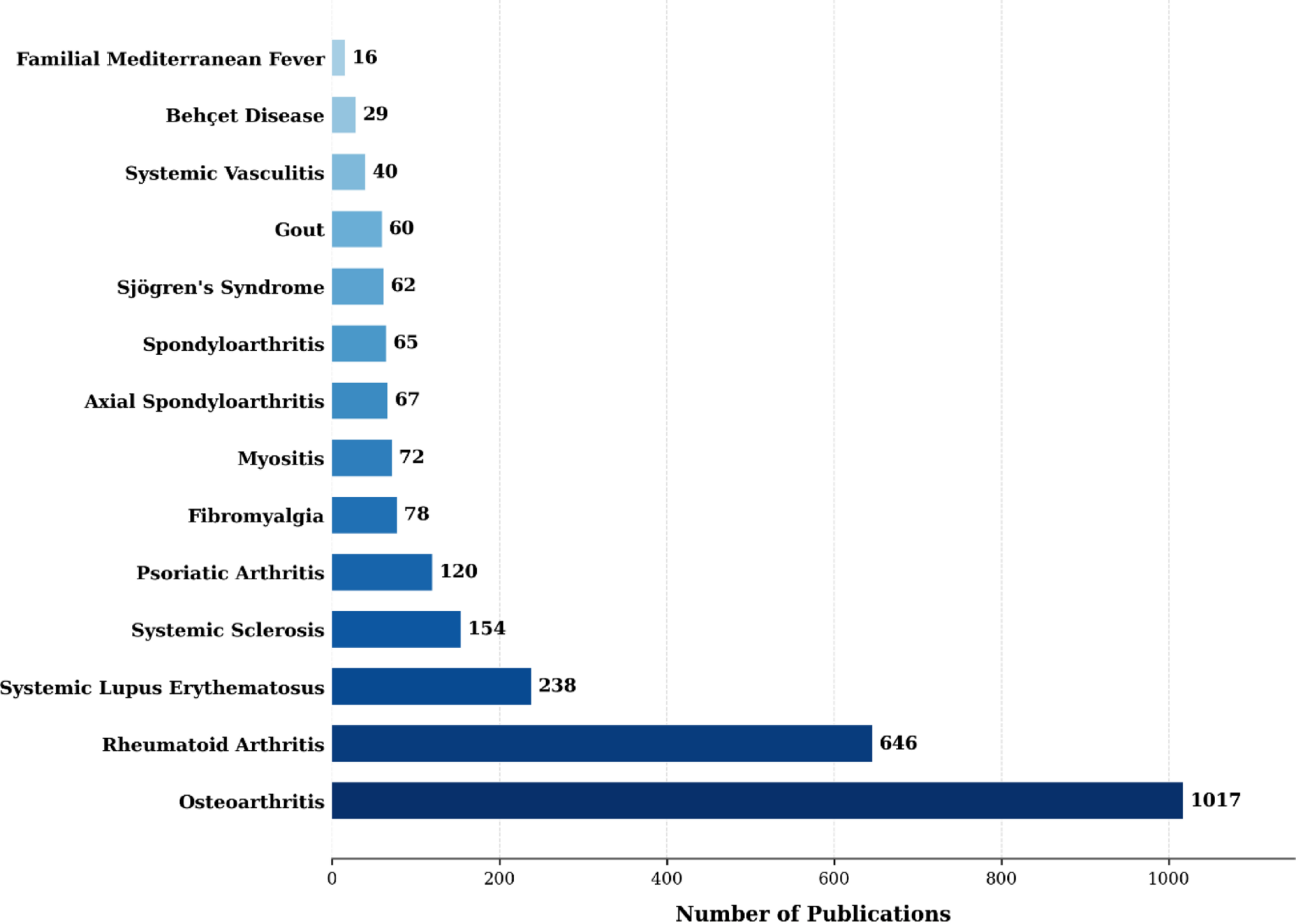
Distribution of artificial intelligence-related publications by rheumatic disease. Each disease category was searched independently in Scopus. Because some studies address multiple conditions, the total number of publications across all diseases exceeds the overall dataset of 1,057 articles

Analysis of disease-specific distributions showed that osteoarthritis (n = 1,017) and rheumatoid arthritis (n = 646) accounted for the majority of AI-related articles. Systemic lupus erythematosus (n = 238) and systemic sclerosis (n = 154) followed as the next most researched disorders. Psoriatic arthritis (n = 120), fibromyalgia (n = 78), myositis (n = 72), axial spondyloarthritis (n = 67), spondyloarthritis (n = 65), Sjögren syndrome (n = 62), and gout (n = 60) formed a mid-tier group with moderate research activity. In contrast, systemic vasculitis (n = 40), Behçet disease (n = 29), and familial Mediterranean fever (n = 16) were minimally represented in the AI literature, highlighting underexplored areas in the field (Fig. 3).

### Survey-based studies

Eleven survey-based studies were identified from 2022 to 2025. Most were published in 2024 (n = 5) and 2025 (n = 4). The majority of survey reports were published in rheumatology journals, particularly *Rheumatology International* (n = 3). Target populations included rheumatology professionals (n = 4), patients with rheumatic diseases (n = 4), and mixed or pediatric-specific groups (n = 3). Table 5 summarizes these findings.

**Table 5:** Survey-based studies on artificial intelligence and rheumatology

No	Title	Target Population / Topic	Journal	Year	Citations
1	Patient's Perception of Digital Symptom Assessment Technologies in Rheumatology: Results From a Multicentre Study	Patient perceptions of AI-based digital symptom assessment tools in rheumatology (multicentre)	*Frontiers in Public Health*	2022	35
2	Perception of Chat Generative Pre-trained Transformer (Chat-GPT) AI tool amongst MSK clinicians	MSK clinician perceptions and attitudes toward ChatGPT as a clinical AI tool	*Journal of Clinical Orthopaedics and Trauma*	2023	18
3	Artificial intelligence in rheumatology: perspectives and insights from a nationwide survey of U.S. rheumatology fellows	AI knowledge, attitudes, and perceived barriers among U.S. rheumatology trainees	*Rheumatology International*	2024	6
4	Artificial intelligence in rheumatology: status quo and quo vadis — results of a national survey among German rheumatologists	Current AI use and perceived opportunities/barriers among German rheumatologists	*Therapeutic Advances in Musculoskeletal Disease*	2024	7
5	Prediction of the acceptance of telemedicine among rheumatic patients: a machine learning-powered secondary analysis of German survey data	ML-based prediction of telemedicine acceptance using patient survey data	*Rheumatology International*	2024	7
6	Doctor Versus Artificial Intelligence: Patient and Physician Evaluation of Large Language Model Responses to Rheumatology Patient Questions in a Cross-Sectional Study	Patient and physician evaluation of LLM chatbot responses to rheumatology questions	*Arthritis and Rheumatology*	2024	52
7	Reliability of a generative artificial intelligence tool for pediatric familial Mediterranean fever: insights from a multicentre expert survey	Expert survey on reliability of generative AI for pediatric FMF management	*Pediatric Rheumatology*	2024	11
8	Patient experiences, attitudes, and profiles regarding artificial intelligence in rheumatology: a German national cross-sectional survey study	Patient perspectives on AI use in rheumatological care (web-based national survey)	*Rheumatology International*	2025	5
9	Knowledge, Attitude, and Practice of Artificial Intelligence (AI) among Rheumatology Professionals: An Online Cross-Sectional Survey	AI awareness, attitudes, and clinical practice patterns among Indian rheumatology professionals	*Mediterranean Journal of Rheumatology*	2025	3
10	Adoption and perception of LLM-based chatbots in health care: An exploratory cross-sectional survey of individuals with rheumatic diseases	Patient adoption and perception of LLM chatbots among individuals with rheumatic diseases	*Rheumatology Advances in Practice*	2025	1
11	Exploring the Acceptance and Opportunities of Using a Specific Generative AI Chatbot to Assist Parents in Managing Pediatric Rheumatological Chronic Health Conditions: Mixed Methods Study	Parental acceptance and opportunities of AI chatbot use in pediatric rheumatology care	*JMIR Pediatrics and Parenting*	2025	3

*AI* artificial intelligence, *LLM* Large Language Model, *MSK* Musculoskeletal, *ChatGPT* Chat Generative Pre-trained Transformer, *FMF* Familial Mediterranean Fever, *ML* Machine Learning

### Retracted items

Two articles were identified as retracted in the dataset. The first, published in the *Journal of Healthcare Engineering* (2022, 5 citations, open access), was retracted in 2023 after the publisher's research integrity team confirmed that the peer review process had been compromised. The second article was published in *Microprocessors and Microsystems* (2021, 3 citations). It was retracted in 2024 due to irregularities in the peer review process for the special issue, as well as a configuration error in the editorial system that bypassed standard oversight.

## Discussion

This cross-sectional bibliometric analysis demonstrates that AI research in rheumatology has been documented since 1982, but experienced substantial growth beginning in 2019, reaching a peak in 2025. Analysis of the ten most frequently used keywords revealed that only rheumatoid arthritis appears among rheumatic diseases, indicating that AI research remains concentrated on specific conditions. This trend aligns with the distribution of publications by disease, as osteoarthritis and rheumatoid arthritis were the most prevalent in the AI literature.

Linear regression analysis confirmed that this increase of publications in the field was statistically significant. Comparable growth patterns have been documented in other clinical disciplines. Bibliometric analyses in cardiology, autoimmune diseases, and rehabilitation demonstrated a significant rise in AI-related publications after 2019–2020 [16–18]. In rheumatology, Polyzou and Baraliakos [19] identified a pronounced upward trend between 2010 and 2024, although they noted that scientific output was concentrated among a limited group of authors. The present study encompassed the entire period from the earliest publication in the literature through 2026, providing a comprehensive overview of the field's historical development. These observed upward trends indicate that the global surge in AI research is also reflected in rheumatology.

Analysis of the distribution by country showed that the United States leads. Previous bibliometric analyses have similarly reported that the United States maintains its leadership in AI research [20, 21]. The high rankings of the United Kingdom and Germany in the current study demonstrate Europe's significant engagement in this domain. India's third-place ranking was particularly notable and may be attributed to its rapidly expanding technology ecosystem and rising academic output. Examination of the distribution by institution showed that US-based institutions are prominently represented at the top, consistent with the country's overall leadership in publishing. The presence of three German institutions among the top six highlighted Germany's concentrated institutional capacity in rheumatology. Combined keyword analysis and disease-based distribution revealed that AI research in rheumatology was concentrated in specific thematic and disease-focused areas. The frequent use of "Machine Learning" as a keyword indicates a methodological shift in the literature toward machine-learning-based approaches [22]. Rheumatoid arthritis ranked highly in both keyword frequency and disease-based distribution, confirming its status as a primary focus of AI research in this field [23]. Furthermore, osteoarthritis emerged as the most extensively studied topic, ranking first by a significant margin in disease-based analysis [24]. Conversely, systemic vasculitides, Behçet disease, and familial Mediterranean fever were underrepresented in the literature, suggesting these diseases remain underexplored in AI research and warrant prioritization in future studies. Analysis of journal-based distribution indicated that publications were primarily concentrated in rheumatology journals. The prominence of *Rheumatology International* in the field suggests a strong receptiveness to AI research within this journal. The presence of *Annals of the Rheumatic Diseases* among the top ten journals also demonstrates that AI research is increasingly represented in leading rheumatology journals.

The predominance of survey-based studies published in 2024–2025 demonstrates that incorporating the rheumatology community's attitudes and experiences regarding AI into the research agenda is a recent development. The concentration of current studies in Germany and the USA underscores the need for data from diverse healthcare systems and geographic regions to enrich the literature. To achieve a more comprehensive understanding of the field, future research should include international surveys involving rheumatology professionals in low- and middle-income countries, physician surveys evaluating training needs and barriers to AI adoption, surveys exploring patient experiences in underrepresented diseases such as Behçet disease and familial Mediterranean fever.

The results of this bibliometric analysis have several practical implications. Standardizing keywords is a critical area for improvement. Authors should use precise, consistent terminology, including the specific names and versions of AI models used in their studies (e.g., ChatGPT-4, Gemini), to improve the reproducibility and accuracy of future literature searches [25]. Enhanced promotion of open-access publishing, combined with the integration of AI tools into specialist rheumatology platforms, could further extend the reach and influence of AI-driven research. As AI applications become more integrated into clinical rheumatology, it is essential to address the associated risks of data security, patient anonymity, and ethical accountability. These issues are especially pertinent when large language models process sensitive patient data and should be explicitly examined in future studies to promote responsible and transparent AI implementation [26].

### Limitations

Several limitations should be acknowledged when interpreting these data. The literature search was restricted to the Scopus database and did not include other reputable databases, such as Medline, Web of Science and the Directory of Open Access Journals. As the search was confined to English documents, studies published in other languages, particularly those from Eastern Europe, Central Asia, and Latin America, may be underrepresented. Although the search term "rheum*" broadly captures rheumatology-related literature, it does not include terms such as "autoimmunity," "immunity," or "inflammation." Including these terms could yield different results, but the wildcard's broad scope suggests the core publication pool is substantially represented.

## Conclusion

This bibliometric analysis demonstrates that AI research in rheumatology has a longstanding history, dating back to 1982, and has experienced substantial growth since 2019. The dominance of the United States, the United Kingdom, and India among leading contributors indicates that research output is geographically concentrated within specific centers. The frequent occurrence of rheumatoid arthritis and osteoarthritis in both keyword and disease-based analyses suggests that these diseases remain primary areas of focus for AI research in rheumatology. In contrast, the limited representation of diseases such as systemic vasculitis, Behçet disease, and familial Mediterranean fever highlights important gaps and priority areas for future research studies. The recent increase in survey-based studies reflects the growing importance of clinician and patient perspectives. Collectively, these findings underscore the need to enhance international collaboration, address research gaps, and strengthen ethical and methodological standards to facilitate broader adoption of AI in rheumatology.

## Data Availability

Raw data can be provided upon reasonable request.
